# N-terminal proteomics of *Mycobacterium marinum* using bottom-up label-free quantitative analysis in data-dependent acquisition mode on a timsTOF Pro mass spectrometer

**DOI:** 10.1128/mra.01263-23

**Published:** 2024-03-13

**Authors:** Daniel D. Hu, Simon D. Weaver, Owen A. Collars, Patricia A. Champion, Matthew M. Champion

**Affiliations:** 1 Department of Chemistry and Biochemistry, University of Notre Dame, Notre Dame, Indiana, USA; 2 Department of Biological Sciences, University of Notre Dame, Notre Dame, Indiana, USA; Department of Biological Sciences, Wellesley College, Wellesley, Massachusetts, USA

**Keywords:** proteomics, N terminal acetylation, acetylation, timsTOF, mycobacteria, virulence determinants, virulence factors

## Abstract

N-terminal acetylation in *Mycobacterium tuberculosis* is correlated with pathogenic activity. We used genomics and bottom-up proteomics to identify protein Emp1 as the sole acetyltransferase responsible for acetylation of EsxA, a known virulence factor. Using custom data analysis, we screened the proteome to identify 22 additional putative substrates of Emp1.

## ANNOUNCEMENT

*Mycobacterium tuberculosis* is the causative agent of human tuberculosis, a disease of global concern (1, 2). *Mycobacterium marinum* is a widely used model for studying ESAT-6 system-1 (ESX-1) protein secretion in *M. tuberculosis* (3–5). EsxA (6-kDa early secretory antigenic target ESAT-6) is a highly conserved secreted protein that is required for mycobacterial pathogenesis (6–9). In both species, EsxA is N-terminally acetylated (10, 11). We used genetics coupled with bottom-up proteomics to identify and verify that Emp1 is the sole enzyme responsible for acetylating EsxA (12).

*C*ell-associated proteins from *M. marinum* M strain (American Type Culture Collection, BAA-535) maintained at −80°C were generated following 48 hours of growth in Sauton’s defined broth at 30°C, described in Collars et al. (12) Protein concentration was measured using the Pierce MicroBCA kit (Thermo Scientific). Protein (100 µg) for each sample was extracted by acetone precipitation. Dried samples were resuspended in 150-mM triethylammonium bicarbonate (TEAB) and 12% sodium dodecyl sulfate. Proteins were reduced in 100-mM tris(2-carboxyethyl)phosphine at 95°C for 10 min then alkylated in 100-mM iodoacetamide for 30 min in darkness. Samples were acidified to 1.2% with H_3_PO_4_ and flocculated with 7× volume of 100-mM TEAB in 90% methanol (binding buffer). Individual samples were loaded and spun through S-Trap Minis (Protifi). Filters were washed (150 µL) twice with binding buffer and once with 1:1 methanol:chloroform. Filters were placed in new collection tubes, and 2 µg of sequencing-grade trypsin (Promega Corporation) in 160-µL 100-mM TEAB was added. Samples were incubated for 4 hours at 37°C. Following digestion, samples were spun down and eluted by two 80-µL additions of 0.1% formic acid (FA) and one of 0.1% FA in 50% acetonitrile. Samples were vacuum concentrated and desalted with Oasis-HLB solid-phase extraction cartridges (Waters) following the manufacturer’s instructions.

Dried samples were resuspended in 0.1% FA to 500 ng/µL. Each sample (1 µL) was injected in triplicate into a Bruker nanoElute and timsTOF Pro system using data-dependent acquisition parallel accumulation serial fragmentation. A 90-min 600-nL/min gradient from 4%–30% (acetonitrile in 0.1% FA) on a 75 µm × 100 mm PepSep C_18_ column was used. CaptiveSpray voltage was set to 1,700 V. tandem mass spectrometry collision energy settings ramped 20 eV at 0.6 ion mobility to 70 eV at 1.6 ion mobility. Instrument parameters were set to default for proteomic studies with the following differences: quadrupole low-mass set to 20 *m*/*z*, pre-pulse storage to 5 μs.

Peptide identification and label-free quantitation were performed using PEAKS:Online Xpro (build 1.4.2020–10-21_171258, Bioinformatics Solutions Inc.). Results were filtered to a false discovery rate of <1%. The database used was an *M. marinum* M strain FASTA file from Mycobrowser (v.4). Carbamidomethylation of cysteines was set as a fixed modification. Variable modifications were protein N-terminal acetylation, deamidation of asparagine and glutamine, oxidation of methionine, and pyroglutamate formation on glutamine and glutamatic acid. Quantitation was normalized to total ion current. All other search parameters were default.

Custom scripting was used for analysis, written in R (v.4.3.0) (13) using Rstudio (build 463) (14). The code can be found on Zenodo (https://doi.org/10.5281/zenodo.10635943). The bio-replicate with the highest number of peptide identifications were analyzed. Briefly, technical replicates within bio-replicates were median normalized to the most intense injection. Filtering was performed to remove peptides with three or more missing values between the six injections of wild-type (WT) and Δ*emp1*/comp. The Δ*emp1* strain was not considered in this filtering step because it was hypothesized to contain missing acetylated peptides. N-terminally acetylated peptides were identified, and technical replicates were averaged. Peptidoforms from the same protein were collapsed using weighted averages by peptide area. k-Means clustering (*k* = 2) was performed to cluster each protein, with the inputs to the clustering being the pairwise ratios of N-terminally acetylated peptide area between all strains. Analysis of variance and Tukey’s statistical tests were performed within the two protein clusters (EsxA-like and other) between the three strains (WT, Δ*emp1*, and Δ*emp1*/comp) using the median-normalized, log_10_-transformed N-terminal-acetylated peptide areas. Missing values from the proteins of interest were imputed using a normal distribution around the limit of quantification, which was determined using the average and standard deviation of the minimum normalized peptide area for each injection.

**Fig 1:**
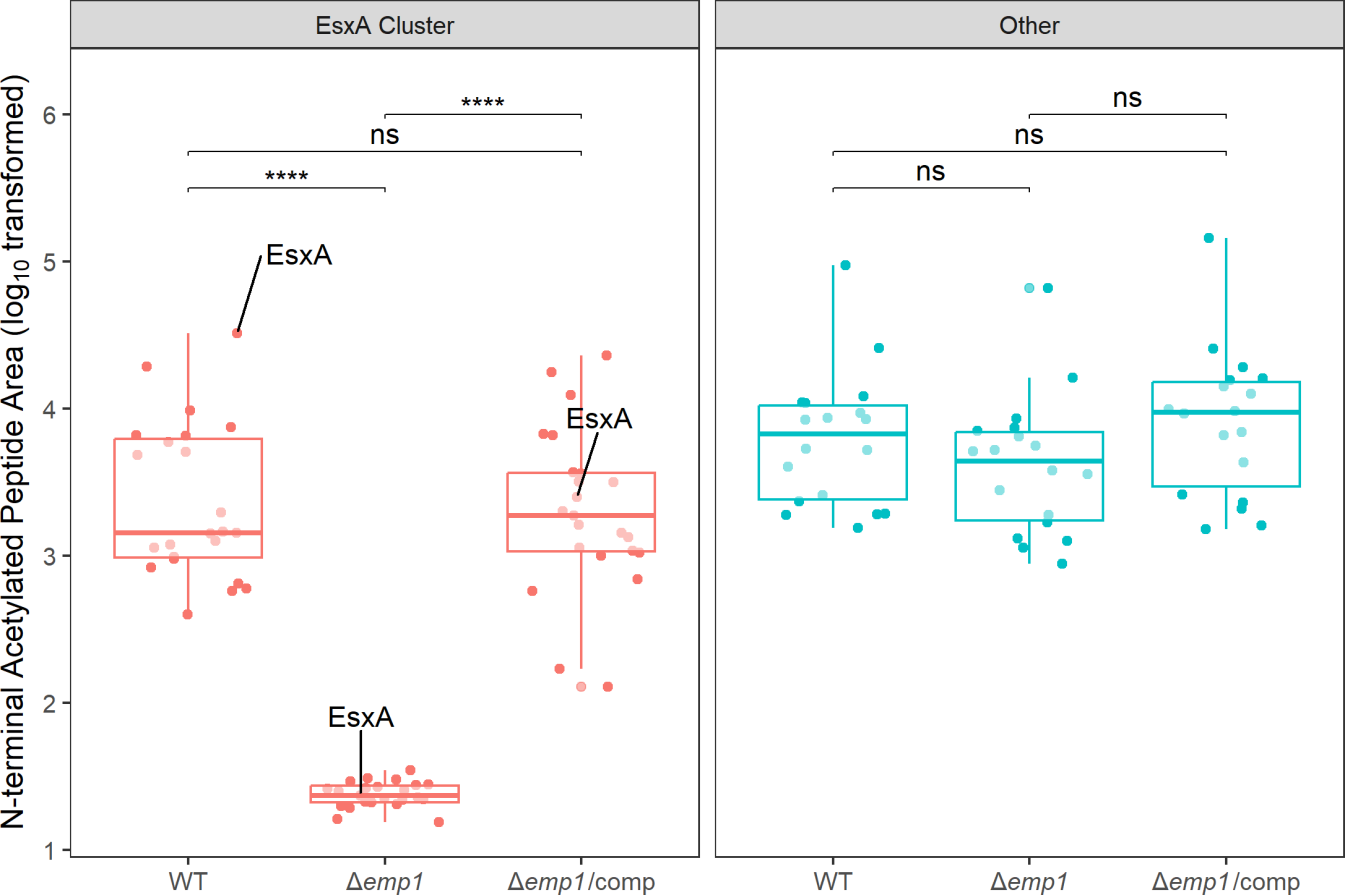
Proteins with measured N-terminally acetylated peptides across three samples and between the two clusters. ****P < 0.0001 by Tukey’s honestly significant difference test. ns, not significant.

The final result of these analyses was a list of proteins that showed N-terminal acetylation patterns similar to EsxA across the WT, Δ*emp1*, and Δ*emp1*/comp strains as shown in Fig. 1. The cluster of proteins that included EsxA shows significantly lower acetylation in the Δ*emp1* strain compared to WT and Δ*emp1*/comp, whereas the other cluster shows no significant differences. Overall, 41 proteins showed measurable N-terminal acetylation levels in the WT and Δ*emp1*/comp strains, 22 of which clustered with EsxA, indicating that they are likely substrates of Emp1. These proteins are listed in Table 1.

**TABLE 1:** Names and accessions of proteins in cluster containing EsxA^*a*^

Accession	Protein name
MMAR_5450	6-kDa early secretory antigenic target EsxA
MMAR_0057	Leucyl-tRNA synthetase LeuS
MMAR_0638	GrpE protein (Hsp-70 cofactor)
MMAR_0879	3-Oxoacyl-[acyl-carrier-protein] synthase III FabH
MMAR_1047	50S ribosomal protein L5, RplE
MMAR_1184	Homoserine O-acetyltransferase MetA
MMAR_1185	O-Acetylhomoserine sulfhydrylase MetC
MMAR_1962	Dihydrodipicolinate synthase DapA
MMAR_1973	Conserved protein
MMAR_2089	Conserved protein
MMAR_2267	Conserved protein
MMAR_2289	3-Oxoacyl-[acyl-carrier protein] reductase, FabG1
MMAR_2442	Conserved protein
MMAR_2469	Argininosuccinate lyase ArgH
MMAR_2527	Cytidylate kinase, Cmk
MMAR_2568	Carbohydrate phosphorylase
MMAR_2821	Isocitrate lyase AceAb
MMAR_3645	cysteine synthase a CysK1
MMAR_4135	carbonic anhydrase
MMAR_4553	ATP-dependent DNA helicase II UvrD1
MMAR_4871	Conserved hypothetical protein
MMAR_5149	Phosphoserine phosphatase SerB
MMAR_5350	Conserved hypothetical protein

^*a*^ N-terminal acetylation intensity is reduced in the ∆emp1 sample compared to WT and ∆emp1/comp. This table is excised from a larger supplemental information from Collars et al. (12).

## Data Availability

Raw and processed data are available through MassIVE and PrideDB 81 (ftp://MSV000091442@massive.ucsd.edu and ftp://massive.ucsd.edu/MSV000091442 Pride Accession: PXD040693).
